# Doctors Disagree

**Published:** 1886-05

**Authors:** 


**Doctor's Disagree.**—We clip the two following items from the May 12th, issue of the New York *World*, and reproduce them verbation.

[*Dr. William Lohman, of Baltimore*].

I don't believe in Pasteur's inoculation theory because I don't believe in hydrophobia. It is in my opinion an imaginary disease, and I defy anybody to produce a well-authenticated instance where hydrophobia attacked an idiot or an infant bitten by a rabid dog. It needs a good, vivid imagination as an adjunct of the disease. Some years ago a man came to me for cauterization of what he claimed was the bite of a mad dog. It did not look to me like a wound made by teeth, but I cauterized it to satisfy him. A month afterwards that man died with all the symptoms of hydrophobia as described by standard authorities. After his death it was established conclusively that the wound was made by a nail in the fence that he had climbed to get away from the dog, and also the animal was very old and only had three teeth—those very far back in the jaw and impossible to use to bite with. That case shattered my faith in hydrophobia, and subsequent investigations destroyed it altogether. There is no such thing.

Little Rock Ark.—A dog belonging to A. J. Hall went mad last week, and among the animals which it wounded in its wandering about the farm was a milch cow. The cow showed no signs of being affected by the wound. Yesterday, however, the animal began showing symptoms of hydrophobia, and at the same time the farmer's two little children, who had been nourished with the cow's milk, exhibited similar symptoms, and are in a crytical condition, suffering the most terrible agonies. The other members of the family are also ill, but their symptoms are not alarming,and hope is expressed that they may recover.

It would appear from the above that there *is* such a disease as *hydrophobia*, or that that deluded cow was gifted with a most vivid imagination, which she was ingenious enough to communicate to the precotious little ones, nourished by her lecteal bounty.

What is one to conclude when these favored M. D's, Differ so widely in opinion:

Like the boy at the show, "you pays your money and you takes your choice."

